# Different scan areas affect the detection rates of diabetic retinopathy lesions by high-speed ultra-widefield swept-source optical coherence tomography angiography

**DOI:** 10.3389/fendo.2023.1111360

**Published:** 2023-02-20

**Authors:** Mengyu Li, Mingzhu Mao, Dingyang Wei, Miao Liu, Xinyue Liu, Hongmei Leng, Yiya Wang, Sizhu Chen, Ruifan Zhang, Yong Zeng, Min Wang, Jie Li, Jie Zhong

**Affiliations:** ^1^ Department of Ophthalmology, Sichuan Provincial People’s Hospital, University of Electronic Science and Technology of China, Chengdu, China; ^2^ Department of Ophthalmology, Chinese Academy of Sciences Sichuan Translational Medicine Research Hospital, Chengdu, China; ^3^ Eye School, Chengdu University of Traditional Chinese Medicine, Chengdu, China; ^4^ School of Medicine, University of Electronic Science and Technology of China, Chengdu, China; ^5^ Department of Ophthalmology, Dayi Shaoxiang Hospital, Chengdu, China

**Keywords:** diabetic retinopathy, microaneurysms, intraretinal microvascular abnormalities, retinal neovascularization, swept-source optical coherence tomography angiography, capillary non-perfusion areas

## Abstract

**Introduction:**

The study aimed to determine the effect of the scanning area used for high-speed ultra-widefield swept-source optical coherence tomography angiography (SS-OCTA) on the detection rate of diabetic retinopathy (DR) lesions.

**Methods:**

This prospective, observational study involved diabetic patients between October 2021 and April 2022. The participants underwent a comprehensive ophthalmic examination and high-speed ultra-widefield SS-OCTA using a 24 mm × 20 mm scanning protocol. A central area denoted as “12 mm × 12 mm-central” was extracted from the 24 mm × 20 mm image, and the remaining area was denoted as “12 mm~24mm-annulus.” The rates of detection of DR lesions using the two scanning areas were recorded and compared.

**Results:**

In total, 172 eyes (41 eyes with diabetes mellitus without DR, 40 eyes with mild to moderate non-proliferative diabetic retinopathy (NPDR), 51 eyes with severe NPDR, and 40 eyes with proliferative diabetic retinopathy (PDR) from 101 participants were included. The detection rates of microaneurysms (MAs), intraretinal microvascular abnormalities (IRMAs), and neovascularization (NV) for the 12 mm × 12 mm central and 24 mm × 20 mm images were comparable (p > 0.05). The detection rate of NPAs for the 24 mm × 20 mm image was 64.5%, which was significantly higher than that for the 12 mm × 12 mm central image (52.3%, p < 0.05). The average ischemic index (ISI) was 15.26% for the 12 mm~24mm-annulus, which was significantly higher than that for the 12 mm × 12 mm central image (5.62%). Six eyes had NV and 10 eyes had IRMAs that only existed in the 12 mm~24mm-annulus area.

**Conclusions:**

The newly developed high-speed ultra-widefield SS-OCTA can capture a 24 mm × 20 mm retinal vascular image during a single scan, which improves the accuracy of detecting the degree of retinal ischemia and detection rate of NV and IRMAs.

## 1 Introduction

Diabetic retinopathy (DR), one of the most common microvascular complications of diabetes, is among the main causes of vision loss among the working-age population globally. Several meta-analyses have predicted that the global prevalence of diabetes will be 600–700 million people in the next two decades (1, 2). Another study reported an estimated prevalence of 34.6% for all forms of DR and 7.0% for proliferative DR (PDR) (3). PDR is the leading cause of vision loss and preventable blindness in the working-age population.

Within the past decade, several novel imaging technologies have been developed for screening and diagnosing DR. These include laser scanning fundus imaging and swept-source optical coherence tomography angiography (SS-OCTA). As a noninvasive alternative, SS-OCTA has been used in examinations for retinal neovascular diseases including DR. Previous studies have demonstrated that OCTA can detect vascular lesions in DR, such as microaneurysms (MAs), intraretinal microvascular abnormalities (IRMAs), neovascularization (NV), capillary non-perfusion areas (NPAs), hard exudates (HEs), and diabetic macular edema (DME) (4–6).

However, most of the commonly used commercial SS-OCTA devices used in previous studies had a scan area of 12 mm × 12 mm or less centered on the fovea by a single scan. Due to the limitation of the range of observation, clinically significant information outside the scan area may not be captured. Several studies have reported that the imaging area can be increased by stitching images together after multiple scans or adding dioptric lenses to expand the field of view (FOV) (7–10). However, these scanning protocols or techniques required longer durations of acquisition and may introduce more artifacts (8, 10).

The recently developed TowardPi high-speed ultra-widefield SS-OCTA systems have an A-scan rate of 400 kHz (BMizar), which can capture an area of 24 mm × 20 mm (about 120° FOV) in a single scan. In this study, we used the latest high-speed ultra-widefield SS-OCTA to obtain a 24 mm × 20 mm retinal blood flow image and compared its detection of DR lesions detection with a relatively narrower 12 mm × 12 mm image.

## 2 Methods

### 2.1 Participants

This prospective observational study involved patients with DR or diabetes mellitus recruited from the ophthalmic outpatient department of Sichuan Provincial People’s Hospital from October 2021 to April 2022. A total of 172 eyes from 101 participants were included; the participants were 56.50 ± 10.58 (25–86) years old. The durations of diabetes (since diagnosis) among the participants ranged from 1 month to 32 years (**Table 1**). This study was approved by the Ethics Committee of Sichuan Provincial People’s Hospital. Informed consent was obtained from the patients before the examination. All procedures were conducted following the guidelines of the Declaration of Helsinki.

**Table 1:** Demographic characteristics of participants.

Participants (eyes)	101 (172)
Mean ± SD age, y	56.50 ± 10.58
Males/Females	55/46
Type of diabetes (participants)	
Type 1	1
Type 2	100
Mean ± SD duration of diabetes, y	9.66 ± 6.97
Groups, the severity of DR eyes	
No DR in DM patients	41
Mild and Moderate NPDR	40
Severe NPDR	51
PDR	40
Right eyes/Left eyes	87/85
DME eyes	63

DM, diabetes mellitus; DR, diabetic retinopathy; NPDR, non-proliferative diabetic retinopathy; PDR, proliferative diabetic retinopathy; DME, diabetic macular edema.

### 2.2 Inclusion and exclusion criteria

The inclusion criterion was diabetes mellitus diagnosed with or without DR. The exclusion criteria were as follows: uncooperative patients with extremely poor visual acuity or inability to fix the bulbus oculi and severe opacity of the refractive media; vitreoretinopathy caused by other eye diseases; complications other than hypertension, dyslipidemia, and abnormal renal function; intraocular surgery other than for cataracts; poor images, including images with a system built-in automatic quality score below 6/10 (5), images with severe motion artifacts preventing accurate analysis, and blurry images (11); and opacities of the refractive media affecting more than 30% of OCTA images (**Supplementary SFig. 1**) (12).

### 2.3 Examination procedure

All patients underwent a comprehensive ophthalmic examination, including best-corrected visual acuity, intraocular pressure, computerized optometry, fundus photography, ocular biometry, and high-speed ultra-widefield OCTA. We used TowardPi high-speed ultra-widefield SS-OCTA systems (BM-400K BMizar, TowardPi Medical Technology; Beijing, China) to examine the patients. The instrument used a swept frequency laser with a center wavelength of 1,060 nm and a scanning speed of 400,000 times/s. With patient cooperation and no tracking, a single image was obtained in 15 seconds with a scan depth of 6 mm and a FOV of 120° resulting in a maximum fundus imaging area of 24 mm × 20 mm.

The participants were instructed to look at the inner marker light, and a 24 mm × 20 mm SS-OCTA image was obtained with the fovea as the center. Image quality was automatically rated on a scale of 1 to 10 by built-in software. If the image quality is poor (<6/10), the process was repeated and new images were obtained when the image quality was poor; images with better quality were selected for the analysis. For each participant, SS-OCTA was performed using the 24 mm × 20 mm scanning mode, and 76 eyes from 38 patients were also scanned using 12 mm × 12 mm scanning mode at the same time.

### 2.4 Image analysis

The images were annotated by two experienced ophthalmologists. Disagreements between them were openly adjudicated by an independent senior retina specialist (ZL) who had more than 30 years of working experience in diagnosing and treating DR. All lesions were identified based on their characteristics on high-speed ultra-widefield SS-OCTA images, as in previous reports (6, 10, 13). MAs were defined as moderate or hyperreflective spots, and they had various morphologic patterns, including fusiform, saccular, curved, and rarely coiled shapes, in the SS-OCTA images (14). Adjacent to the NPAs, the IRMAs appeared as tortuous, dilated, and annular abnormal microvessels in the retina. After segmentation error correction, the NVs were observed as extraretinal vessels present on the vitreoretinal interface slab. The NPAs were defined as absence of capillary beds between a terminal arteriole and a proximal venule or larger vessel (15), and potential NPAs with areas less than 0.2 mm² were not delineated (16). The hard exudates appeared on the B-scan as bright hyperreflective lesions with posterior shadows (10).

**Figure 1:**
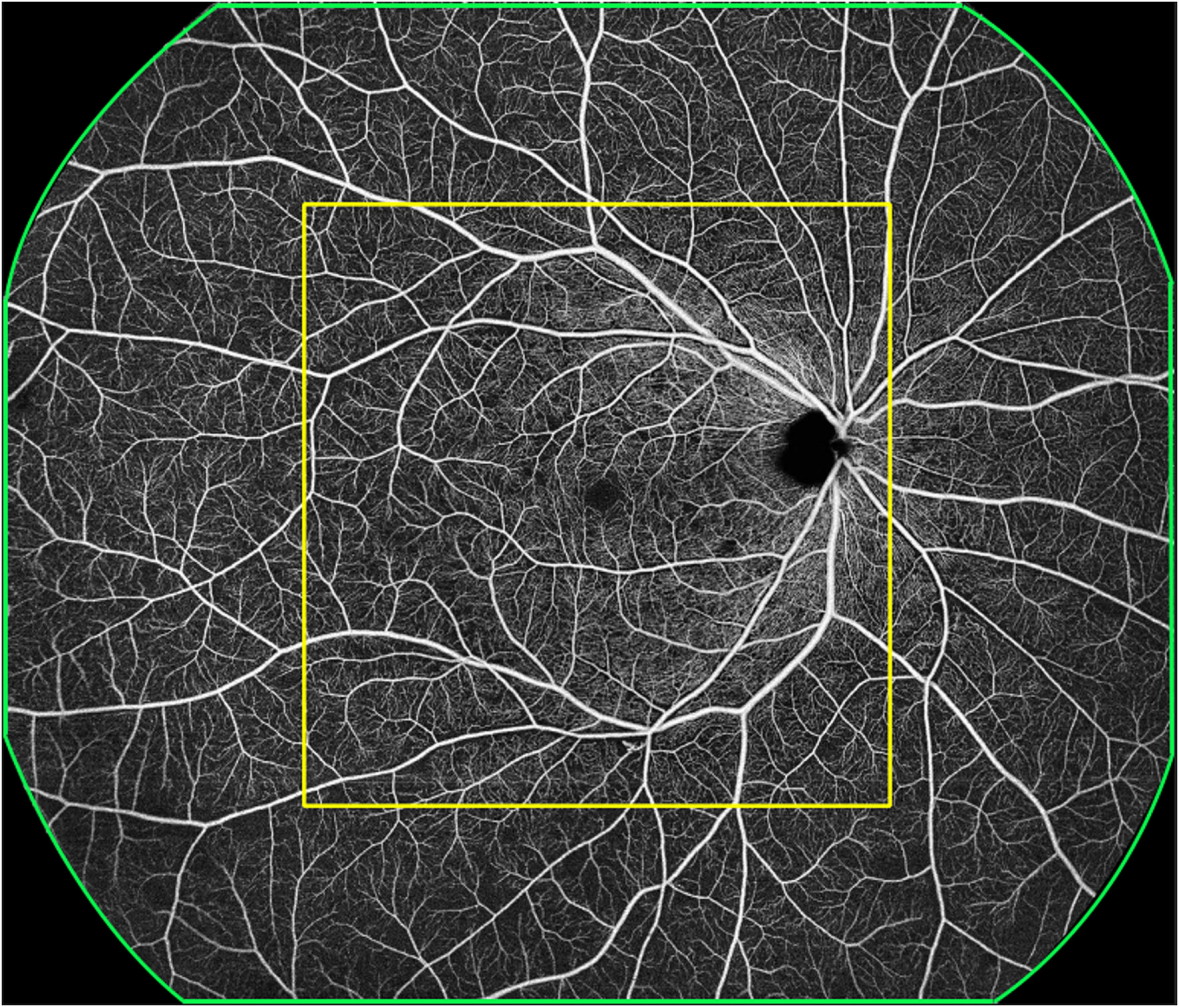
A representative OCTA image. The green outline represents the 24 mm × 20 mm scanning area, with the imaging centered on the fovea. The yellow outline represents the 12 mm × 12 mm scanning area, with the imaging centered on the fovea; this was termed the 12 mm × 12 mm central. The area outside the yellow outline and within the green outline was termed 12 mm~24mm-annulus.

**Figure 2:**
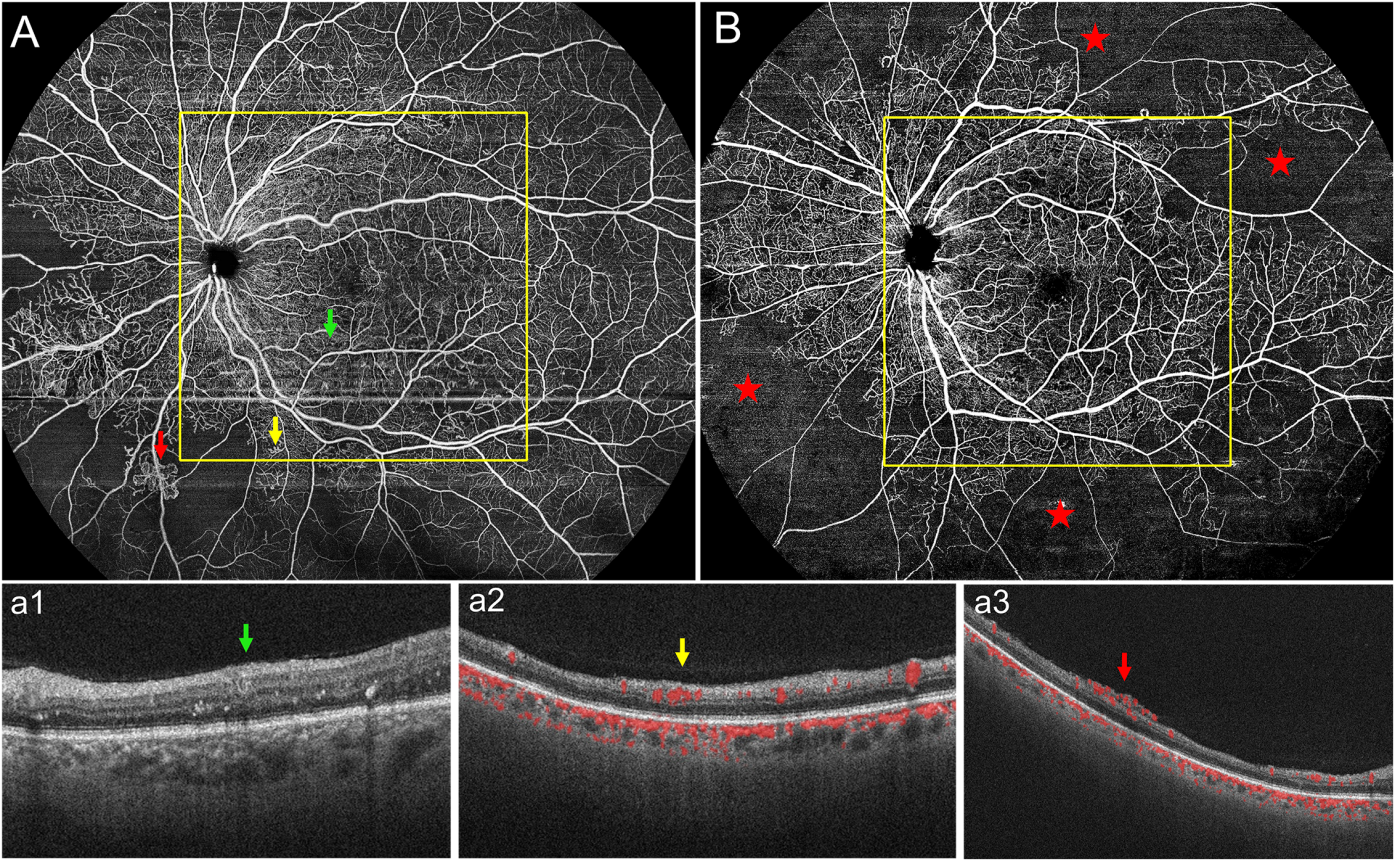
**(A)** Representative 24 mm × 20 mm OCTA image of one eye with PDR. Representative DR lesions are marked with green arrows for microaneurysms (MAs) with corresponding B-scans (a1); yellow arrows for intraretinal microvascular abnormalities (IRMAs) with corresponding B-scans (a2); and red arrows for neovascularization (NV) with corresponding B-scans (a3). **(B)** In another representative 24 mm × 20 mm OCTA image, the red stars represent non-perfusion areas (NPAs); most of the NPAs were seen in the 12 mm~24mm-annulus. PDR, proliferative diabetic retinopathy.

Each 24 mm × 20 mm SS-OCTA image was marked with a 12 mm × 12 mm square centered on the macula with the built-in tool and divided into 12 mm × 12 mm-central and 12 mm~24mm-annulus areas (**Figure 1**). The presence or absence of MAs, IRMAs, NV, NPAs, HEs, and DME was marked in each area (**Figure 2**). The dimensions of the NPAs (mm^2^) were also recorded (16). The ischemic Index (ISI) was calculated by dividing NPAs by the corresponding retinal area (17). To evaluate the accuracy of the 12 mm × 12 mm-central scanning area, which was obtained partially from an area of 24 mm × 20 mm, 76 eyes were selected for both the 12 mm × 12 mm and 24 mm × 20 mm high-speed ultra-widefield SS-OCTA scans.

In this study, severity of DR was graded using the Optos ultra-widefield retinal image (Optos PLC; Scotland, United Kingdom) of all participants (18, 19), according to the International Clinical Diabetic Retinopathy Severity Scale (20). Based on the lesion characteristics, we classified DR into DM without DR, mild to moderate non-proliferative diabetic retinopathy (NPDR), severe NPDR, and proliferative diabetic retinopathy (PDR).

### 2.5 Data analysis

Statistical analysis was performed using SPSS version 26.0 (IBM, Armonk, New York). The data were expressed as mean ± standard deviation. The non-normally distributed data were analyzed using nonparametric tests. The chi-squared test was used to compare the rates of detection of the DR features in different modes. Two-tailed p-values of < 0.05 denoted statistical significance.

## 3 Results

### 3.1 Demographics

In total, 172 eyes (41 eyes with DM without DR, 40 eyes with mild to moderate NPDR, 51 eyes with severe NPDR, and 40 eyes with PDR) from 101 participants were included in the study. The average age of the participants was 56.50 ± 10.58 years, and the average duration since the diagnosis of diabetes was 9.66 ± 6.97 years. A total of 63 participants had DME (**Table 1**).

### 3.2 Detection rates of DR lesions in different areas of a 24 mm × 20 mm high-speed ultra-widefield SS-OCTA image

The detection rates (the number of eyes found divided by the total number of eyes examined) of MAs, IRMAs, and NV were 66.9%, 44.2%, and 19.2% for the 12 mm × 12 mm-central images and 74.4%, 51.2%, and 23.3% for the 24 mm × 20-mm images, respectively (p > 0.05). The detection rate of NPAs was 64.5% for the 24 mm × 20 mm image, which was higher than that for the 12 mm × 12 mm-central image (52.3%) (p < 0.05). The two scan protocols had identical detection rates for HE and DME (p = 1.00, **Table 2**).

**Table 2:** DR lesion detection rates of 12 mm × 12 mm central and 24 mm × 20 mm OCTA images.

	Eyes with DR Lesions present in different scan areas (n/N Eyes, %)		
DR lesions	12 mm × 12 mm central	24 mm×20 mm	p-value
MAs	115/172 (66.9%)	128/172 (74.4%)	.155
IRMAs	76/172 (44.2%)	88/172 (51.2%)	.235
NV	33/172 (19.2%)	40/172 (23.3%)	.429
NPAs	90/172 (52.3%)	111/172 (64.5%)	.029
HE	110/172 (64.0%)	110/172 (64.0%)	1.000
DME	63/172 (36.6%)	63/172 (36.6%)	1.000

MAs, microaneurysms; IRMAs, intraretinal microvascular abnormalities; NV, neovascularization; NPAs, non-perfusion areas; HE, Hard exudates; DME, diabetic macular edema; p-value. The difference was statistically significant (p<.05).

### 3.3 Distribution of nonperfusion areas and ischemic index in different retinal zones

NPAs were present in 111 eyes. The average dimension of the NPAs was 8.09 ± 13.58 mm^2^ in the 12 mm × 12 mm-central image, with an average ISI of 5.62 ± 9.43%. The average dimension of the NPAs was 45.86 ± 58.69 mm^2^ in a 12 mm~24mm-annulus image, with an average ISI of 15.26 ± 19.52% (p < 0.05, **Table 3**).

**Table 3:** ISIs for 12 mm × 12 mm central and 12 mm~24mm-annulus imaging.

	Different scan areas		
DR lesions	12 mm × 12 mm central	12 mm~24mm-annulus	p-value
NPAs area (mm^2^)	8.09 ± 13.58	45.86 ± 58.69	.000
Average ISI (%)	5.62 ± 9.43	15.26 ± 19.52	.000

Data were presented as means ± standard deviations; NPAs, non-perfusion areas; ISI =ischemic index (ISI =NPAs/retinal area); p-value. The difference was statistically significant (p<.05); 12 mm × 12 mm central retinal area is 144 mm^2^, and 12 mm~24mm-annulus retinal area is 300.6 mm^2^.

## 4 Discussion

In our study, we compared the detection rates and distributions of DR lesions in SS-OCTA images with fields of 24 mm × 20 mm (FOV 120°) and 12 mm × 12 mm (FOV 50°). With the increase in FOV, more IRMAs and NV were detected by the ultra-widefield SS-OCTA. The retinal ischemia was more severe in the mid-peripheral retina than in the posterior area. In addition, there was no loss of details of the posterior DR lesions in the 24 mm × 20 mm scanning mode relative to the 12 mm × 12 mm scanning mode.

Within the last decade, SS-OCTA has been gradually used to detect and research vascular changes in DR. Most previous studies used SS-OCTA with a scanning area of 12 mm × 12 mm (FOV 50°) (21–24). Higher FOV (90°) can be achieved using the montage technique. The montage technique allows a higher FOV. However, it has certain limitations, such as misalignment, motion artifacts, and longer processing times (25). Another simple technique for increasing the scan length for OCTA is an extended field imaging technique. However, it leads to a decrease in image resolution and missing retinal vascular information (26). In this study, we used the newly developed and commercialized high-speed ultra-widefield SS-OCTA systems. Its 24 mm × 20 mm scanning mode (444.6 mm^2^) provides a 208.7% larger scanning area than the 12 mm × 12 mm scanning mode (144 mm^2^) and facilitated a wider retinal field (approximately 120° FOV). According to the recommendation of the International Widefield Imaging Study Group, the FOV of the OCTA system in our study can be categorized as ultra-widefield (approximately 110–220°, anterior edge of vortex vein ampulla, and beyond to pars plana) (27).

We evaluated the presence or absence of DR lesions including MAs, IRMAs, NV, NPAs, HE, and DME. The larger scanning area did not increase the detection rate of MAs, IRMAs, and NV (p > 0.05, **Table 2**). However, the detection rate of the NPAs, an indicator of retinal ischemia, increased with the expansion of the scanning area (p < 0.05, **Table 2**). We quantified the NPAs and evaluated the ISI for different areas. The average ISI for the 12 mm × 24 mm-annulus image was 15.26 ± 19.52%, which was significantly higher than that for the 12 mm × 12 mm-central image (5.62% ± 9.43%) (**Table 3**). These results suggested that ischemia was more severe in the peripheral region than in the posterior pole within the 24 mm × 20 mm area (**Figure 2B**). Fan et al. (17) proposed that ISI increases with an increase in the distance from the foveal center. Wang et al. (7) used an OCTA montage image composed of five 12 mm × 12 mm OCTA images for analysis and reported that the capillary non-perfusion of the 50–100° FOV sector in DR was more prominent in the peripheral regions of the retina compared to that of the central FOV sectors. These findings were similar to our results. However, the authors were aware of the several limitations of their study: the 12 mm × 12 mm scans only provide a lateral resolution of 24 μm; the automated montage feature failed at times due to the disparate overlap of the 12 mm × 12 mm scans requiring time-consuming manual correction; and the image distortion may have affected the measurements of peripheral capillary nonperfusion (7). In our study, a single scanning could provide a 24 mm × 20 mm image (FOV 120°) with a transverse resolution of 10 μm and an axial optical resolution of 3.8 μm, which enabled us to observe the capillary non-perfusion areas more accurately. The display of the ischemic area was more comprehensive and intuitive with a wider SS-OCTA image. Theoretically, these non-invasive wider SS-OCTA images could be quickly and repeatedly acquired from the patients for follow-up of the change in capillary drop-out in clinical practice.

**Figure 3:**
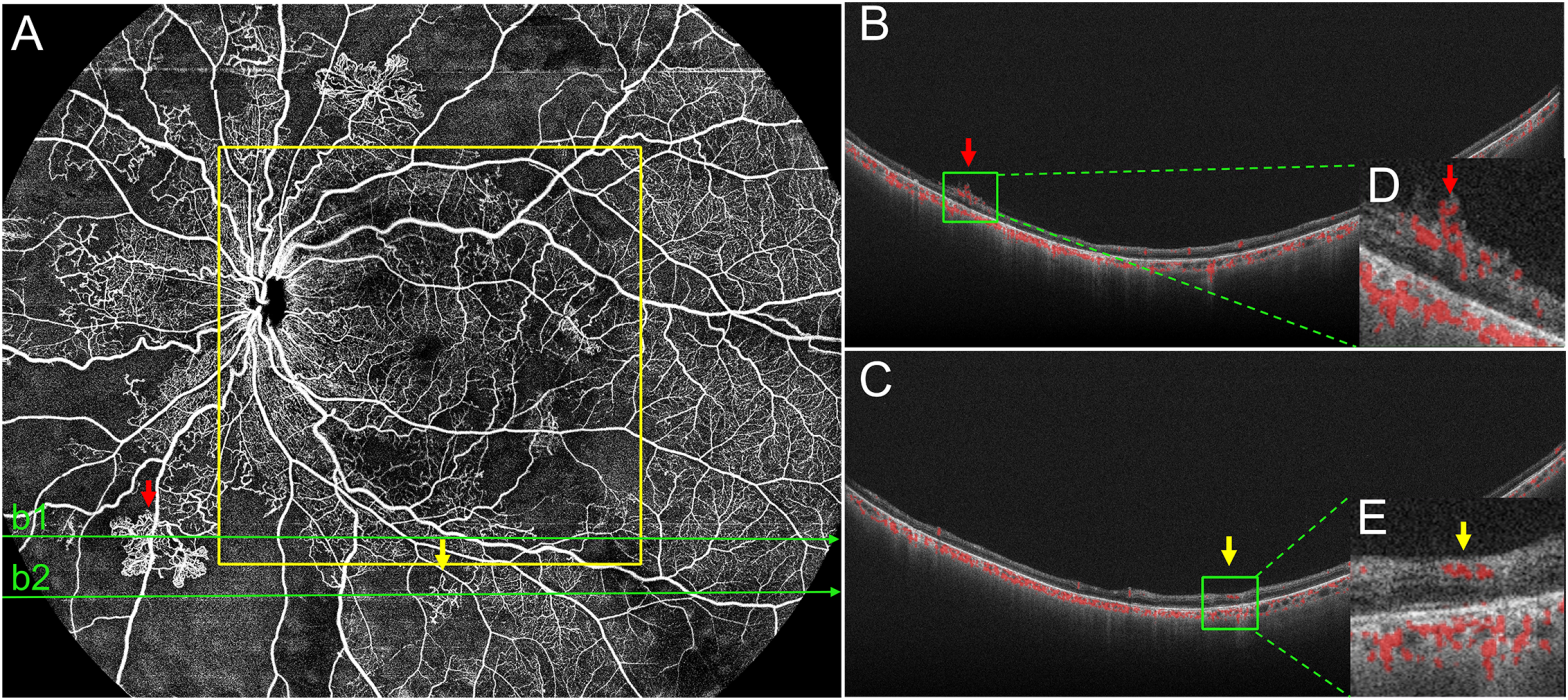
Representative OCTA image of PDR. **(A)** In the 24 mm ×20 mm image, the red arrows mark the neovascularization (NV), and a corresponding BScan image (b1) is provided **(B, D)**. Yellow arrows mark intraretinal microvascular abnormalities (IRMAs) and a corresponding B-Scanimage (b2) is provided **(C, E)**. Structurally, IRMAs were defined as new vessels formed within the retinal layers whereas, retinal NV breached the internal limiting membrane. In this eye, both NV and IRMAs were outside the 12 mm ×12 mm area (yellow outline), but they were accurately detected using the 24 mm ×20 mm scanning mode. PDR, proliferative diabetic retinopathy.

Besides NPAs, IRMAs and NV were vital considerations for DR management. It is important to distinguish between IRMAs and retinal NV to determine disease severity and prognosis and make treatment decisions. SS-OCTA can show frontal and cross-sectional analyses, while B-scans help to differentiate IRMA from NV (28, 29). Similar to the methods used by Lee et al. (30) and Cho et al. (31), we differentiated IRMA from NV by analyzing B-scans (**Figures 2**: a2, a3). In this study, there were no statistically significant differences between the detection rates of IRMAs and NV in the 12 mm × 12 mm-central and 24 mm × 20 mm SS-OCTA images. These results were similar to the findings of previous studies. However, we observed more IRMAs and NV within the 24 mm × 20 mm area than within the 12 mm × 12 mm-central area. (**Figure 3**). Of the total of 40 eyes with PDR and 51 eyes with severe NPDR, 6 eyes had NV and 10 eyes had IRMAs that were only observed within the 12 mm~24mm-annulus area. Compared with the 12 mm × 12 mm-central area, more severe lesions were observed in 16 eyes within the 24 mm × 20 mm area due to an increase in the FOV. With the detection of more regions of NV, 6 eyes that were originally diagnosed with severe NPDR based on 12 mm × 12 mm-central images were diagnosed with PDR. Therefore, a wider scanning field for SS-OCTA can facilitate more accurate determination of DR progression and timely interventions and guide clinical diagnosis and treatment. However, prospective studies with larger sample sizes are needed to test this hypothesis.

**Figure 4:**
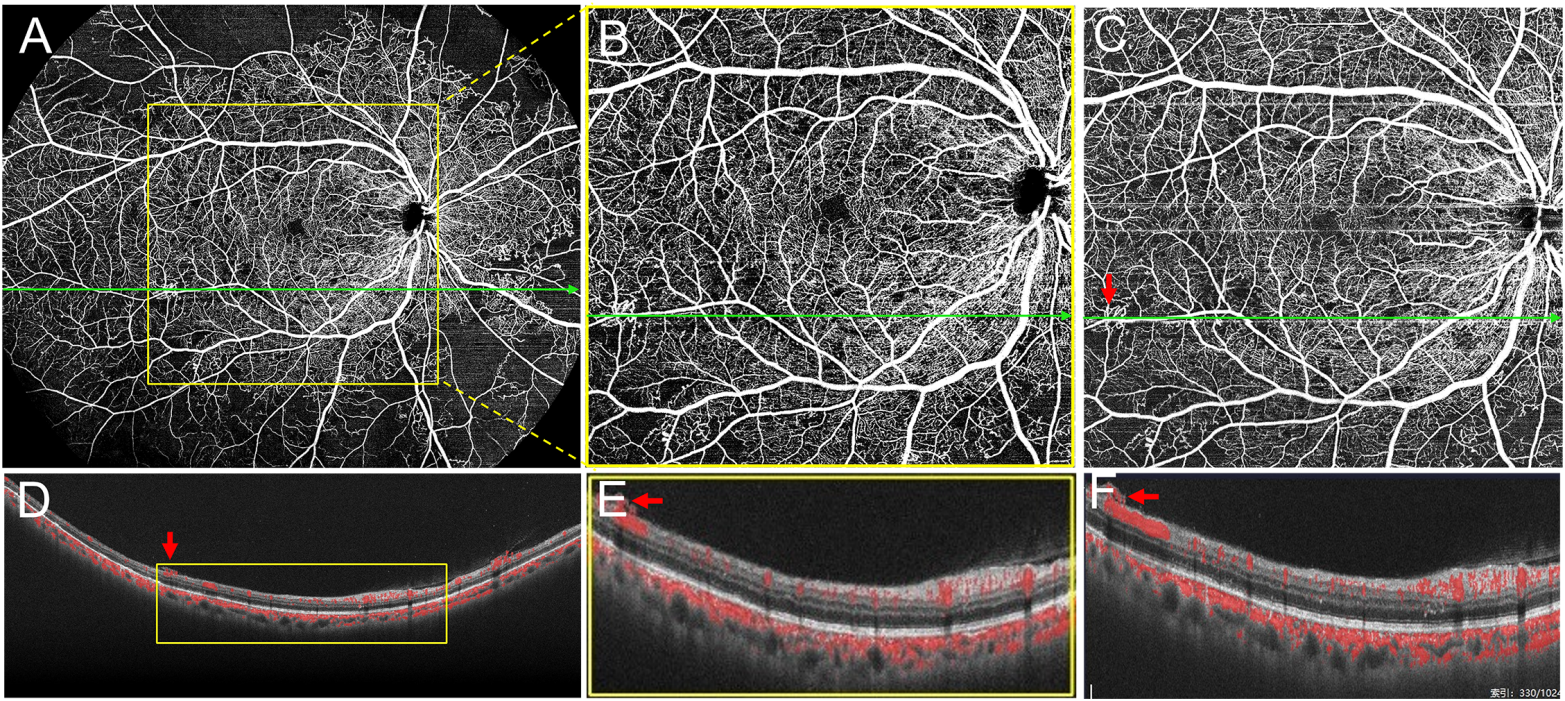
Scans of the same eye using two different protocols. **(A)** The 24 mm ×20 mm scanning mode. **(B)** The 12 mm ×12 mm image was extracted from the yellow outline area in **(A)**. **(C)** Image obtained using the 12 mm × 12 mm scanning mode. **(D–F)** B-Scan images acquired with corresponding scan lines in **(A)**, **(B)**, and **(C)**, respectively. As shown in **(D–F)**, both 24 mm ×20 mm and 12 mm ×12 mm scanning modes show the neovascularization breaking through the internal limiting membrane (red arrows).

Additionally, since this is a newly developed SS-OCTA device, we also investigated differences in the lesion detection rate using the 24 mm× 20 mm and 12 mm × 12 mm scan modes. Seventy-six eyes from 38 patients were scanned using the two modes. We compared the 12 mm × 12 mm SS-OCTA images extracted from the 24 mm × 20 mm scanning mode with the image obtained by the 12 mm × 12 mm scanning mode. There were no statistical differences between the detection rates for all the lesions (**Table 4**), and we found that the 24 mm × 20 mm scanning mode did not lose the information within the 12 mm × 12 mm area during DR lesion detection (**Figure 4**); instead, it provided more information in the mid-peripheral retina. However, the 12 mm × 12 mm scan mode has an advantage of a relatively short scan time.

**Table 4:** Comparison of 12 mm×12 mm central and A-single-scan 12 mm×12 mm OCTA images.

	The same areas for different scanning protocols		
DR lesions	12 mm×12 mm central (eyes)	A-single-scan 12 mm×12 mm (eyes)	p-value
MAs	60/76 (78.9%)	63/76 (82.0%)	0.68
IRMA	46/76 (60.5%)	46/76 (60.5%)	1.00
NV	19/76 (25.0%)	19/76 (25.0%)	1.00
NPAs	52/76 (68.4%)	52/76 (68.4%)	1.00
HE	51/76 (67.1%)	51/76 (67.1%)	1.00
DME	35/76 (46.1%)	35/76 (46.1%)	1.00

MAs, microaneurysms; IRMA, intraretinal microvascular abnormalities; NV, neovascularization; NPAs, nonperfusion areas; HE, hard exudates; DME, diabetic macular edema; p-value. The difference was statistically significant (p<.05).

OCTA can non-invasively reveal the structures of multiple layers of the vascular plexus, including the superficial capillary plexus (SCP), intermediate capillary plexus, deep capillary plexus (DCP), and peripapillary radial plexus (PRP) (32, 33). Some previous studies reported that the DCP was more susceptible to ischemia and significantly related to the progression of DR than the SCP (34, 35). However, we did not analyze the distribution of the NPAs in each capillary plexus in this study, since the automatic stratification of the DCP and SCP in the peripheral retina is not as accurate as that in the macular area. Meanwhile, the manual correction of the entire B-scan images is very time-consuming. This limitation needs to be addressed in further studies.

Our study had several other limitations. This was a single-center study involving a relatively small sample. Similar to the previously reported SS-OCTA, TowardPi high-speed ultra-widefield SS-OCTA was influenced by the turbidity of the refractive medium and artifacts. For the 24 mm × 20 mm scanning mode, some optical occlusion or artifacts may be produced in patients with longer and thicker eyelashes, small eyelid fissures, and poor tear film function, which can affect the observation of DR lesions. In addition, patients with macular disease and DME have poor fixation, which may lead to more projection and motion artifacts and affect image quality. These issues need to be considered in future studies.

Despite our limitations, our data suggest the high-speed ultra-widefield SS-OCTA used in this study can obtain a 24 mm × 20 mm OCTA fundus image in a single scan. The high-speed ultra-widefield SS-OCTA can more accurately reveal the degree of retinal ischemia and also detect more NV and IRMAs lesions using the 24 mm × 20 mm compared with the 12 mm × 12 mm scanning area.

## Data availability statement

The raw data supporting the conclusions of this article will be made available by the authors, without undue reservation.

## Ethics statement

The studies involving human participants were reviewed and approved by Ethics Committee of Sichuan Provincial People’s Hospital. The patients/participants provided their written informed consent to participate in this study.

## Author contributions

JZ, JL, MYL, and MM had full access to the data in the study and take responsibility for the integrity of the data and the accuracy of the data analysis. JZ and JL conceived and designed the research. MYL, MM, DW, ML, XL, HL, YW, SC, RZ, YZ, and MW acquired and analyzed the data. MM and MYL reviewed and graded the images. JL, JZ, MYL, and MM wrote the draft of the manuscript. JZ and JL critically reviewed and extensively revised the manuscript. MM and MYL performed the statistical analyses. JZ, JL, YZ, and RZ acquired funding. JZ and JL supervised the study. All authors contributed to the article and approved the submitted version.
