# Supplementary figures and images for: Different scan areas affect the detection rates of diabetic retinopathy lesions by high-speed ultra-widefield swept-source optical coherence tomography angiography
Source: Front Endocrinol (Lausanne). 2023 Feb 20;14:1111360. doi: 10.3389/fendo.2023.1111360 (PMC9986411; doi:10.3389/fendo.2023.1111360)

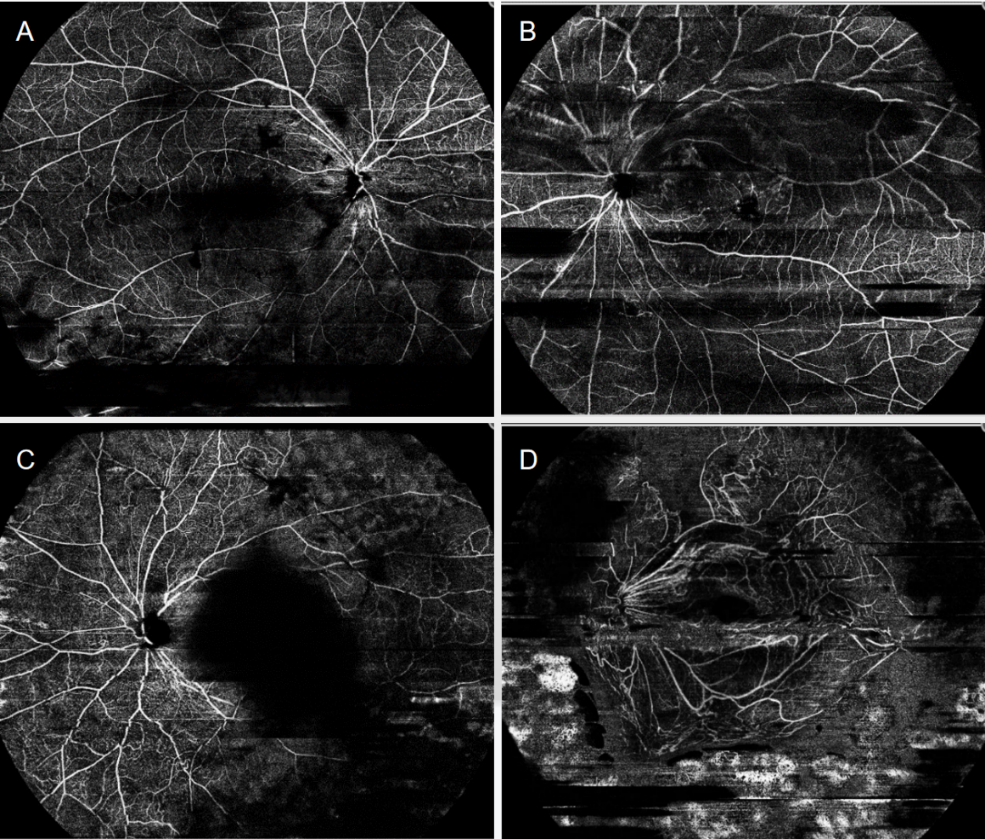

Supplement: Supplementary file 1 [file Image_1.tif]
